# Development of a Pulmonary Nodule Service and Clinical Pathway: A Pragmatic Approach Addressing an Unmet Need

**DOI:** 10.3390/diagnostics15091162

**Published:** 2025-05-02

**Authors:** Georgia Hardavella, Ioannis Karampinis, Nikolaos Anastasiou, Konstantinos Stefanidis, Kyriaki Tavernaraki, Styliani Arapostathi, Nektaria Sidiropoulou, Petros Filippousis, Alexandro Patirelis, Eugenio Pompeo, Panagiotis Demertzis, Stefano Elia

**Affiliations:** 1 6th Department of Respiratory Medicine, “Sotiria” Athens’ Chest Diseases Hospital, 11527 Athens, Greece; 2 Department of Thoracic Surgery, “Sotiria” Athens’ Chest Diseases Hospital, 11527 Athens, Greece; 3 Department of Thoracic Surgery, General Oncology Hospital, “Agioi Anargyroi”, 14564 Kifisia, Greece; 4 Department of Radiology, “Metaxa” Cancer Hospital, 18537 Piraeus, Greece; kostef77@gmail.com; 5 Department of Nuclear Medicine, “Metaxa” Cancer Hospital, 18537 Piraeus, Greece; 6 Imaging and Interventional Radiology Department, “Sotiria” Athens’ Chest Diseases Hospital, 11527 Athens, Greece; 7 Department of Thoracic Surgery, Tor Vergata University Hospital, 00133 Rome, Italy; 8 9th Department of Respiratory Medicine, “Sotiria” Athens’ Chest Diseases Hospital, 11527 Athens, Greece; 9 Department of Medicine and Health Sciences “V.Tiberio”, University of Molise, 86100 Campobasso, Italy

**Keywords:** pulmonary nodules, lung nodules, clinical pathway, pulmonary nodule service

## Abstract

**Background/Objectives:** The surveillance of patients with incidental pulmonary nodules overloads existing respiratory and lung cancer clinics, as well as multidisciplinary team meetings. In our clinical setting, until 2018, we had numerous patients with incidental pulmonary nodules inundating our outpatient clinics; therefore, the need to develop a novel service and dedicated clinical pathway arose. The aims of this study are to 1. provide (a) a model of setting up a novel pulmonary nodule service, and (b) a pragmatic clinical pathway to address the increasing need for surveillance of patients with incidental pulmonary nodules. 2. share real-world data from a dedicated pulmonary nodule service running in a tertiary setting with existing resources. **Methods:** A retrospective review of established processes and referral mechanisms to our tertiary pulmonary nodule service was conducted. We have also performed a retrospective collection and review of data for patients reviewed and discussed in our tertiary pulmonary nodule service between April 2018 and April 2024. **Results:** Our tertiary pulmonary nodule service (PNS) comprises a dedicated pulmonary nodule clinic, a nodule multidisciplinary team (MDT) meeting and a dedicated proforma referral system. Due to the current national health system legislation and relevant processes, patients are required to physically attend clinic appointments. There are various sources of referral, including other departments within the hospital, other hospitals, various specialties in primary care and self-referrals. Between 15 April 2018 and 15 April 2024, 2203 patients were reviewed in the pulmonary nodule clinic (903 females, 1300 males, mean age 64 ± 19 years). Of those patients, 65% (1432/2203) were current smokers. A total of 1365 new patients and 838 follow-up patients were reviewed in total. Emphysema was radiologically present in 72% of patients, and 75% of those (1189/1586) already had a confirmed diagnosis of chronic obstructive pulmonary disease (COPD). Coronary calcification was identified in 32% (705/2203), and 78% of those (550/705) were already known to cardiology services. Interestingly, 27% (368/1365) of the new patients were discharged following their first MDT meeting discussion, and 67% of these were discharged as the reason for their referral was an intrapulmonary lymph node which did not warrant any further action. Among all patients, 11% (246/2203) were referred to the multidisciplinary thoracic oncology service (MTOS) due to suspicious appearances/changes in their nodules that warranted further investigation, and from those, 37% were discharged (92/246) from the MTOS. The lung cancer diagnosis rate was 7% (154/2203). **Conclusions:** The applied pathway offers a pragmatic approach in setting up a service that addresses an increasing patient need. Its application is feasible in a tertiary care setting, and admin support is of vital importance to ensure patients are appropriately tracked and not lost to follow-up. Real-world data from pulmonary nodules services provide a clear overview and contribute to understanding patients’ characteristics and improving service provision.

## 1. Introduction

The detection of incidental pulmonary nodules has significantly increased over the last few years with the wide use of a variety of imaging investigations for numerous conditions, including a range of respiratory cardiac, vascular and colorectal diseases; high-resolution CT scans (HRCT); CT aortograms; CT pulmonary angiograms and CT colonographies. CT angiography, performed for suspicion of pulmonary embolism, is twice as likely to find a new incidental pulmonary nodule or thoracic adenopathy than a pulmonary embolism [1,2]. Similarly, cardiac CT screening for coronary artery disease can incidentally pick up pulmonary nodules in 5–20% of patients [3,4,5], while incidental pulmonary nodules account for 30% of the unsuspected findings of CT urographies offered to investigate hematuria [6].

In addition to the above, the existing and future implementation of lung cancer screening in the US and in other countries, respectively, is anticipated to further increase the detection of pulmonary nodules, and also pose important issues in terms of the further management of suspicious nodules warranting further work-up in a lung cancer pathway, as well as other incidental findings [7,8,9,10,11,12].

This has also been raised in lung cancer screening studies, which although highlighting the importance of lung cancer screening, it showed non-negligible false-positive results in the low-dose CT (LDCT) arm that would require further investigations and/or follow-up [13,14,15]. 

Despite the existing guidelines for the management of pulmonary nodules [16,17,18,19], the increased annual frequency of CT imaging and reporting of incidental pulmonary nodules has overloaded and will further impact the current respiratory services by utilizing more resources and increasing the cost of provided care. Pulmonary nodule management is only going to become more problematic over time; therefore, a formal service provision process should be established [20]. To address this need in our hospital, we have developed a dedicated clinical service and patient pathway for incidental pulmonary nodules. In this paper, we aim to share real-world data from this service and share the model of setting up a pulmonary nodule service, as well as a pragmatic pathway, to address the increasing need for surveillance of patients with incidental pulmonary nodules.

## 2. Materials and Methods 

In this paper, we present the development of a pulmonary nodule service and dedicated patient pathway that was set up at the ‘Sotiria’ Athens Chest Diseases Hospital in Greece in April 2018. ‘Sotiria’ is a National Referral Centre for Respiratory Medicine with >300 dedicated respiratory beds and serves as a central hub for respiratory medicine for both continental Greece and the islands. We have also performed a retrospective review of data for patients that were reviewed and discussed in the ‘Sotiria’ Pulmonary Nodule Service between April 2018 and April 2024.

### 2.1. The Service

By the end of 2017, the surveillance of patients with incidental pulmonary nodules had overloaded our existing lung cancer and general respiratory clinics, and this required several ad hoc discussions with radiologists. At that point, there were numerous such patients (estimated >400) under follow-up by our respiratory services; therefore, the need for developing a novel service and structured clinical pathway arose. The pulmonary nodules service was set up in April 2018, and it consists of a proforma referral system that feeds into a pulmonary nodule clinic and a dedicated multidisciplinary team (MDT) meeting.

According to the national health system’s standard operating procedures and processes, outpatient clinics (including the pulmonary nodule clinic) can only run physically, except for the period of the COVID-19 pandemic and lockdowns, when patients were offered the option of ad hoc virtual consultations. Following the end of the pandemic, initial processes were re-instated, and outpatient clinics (including the pulmonary nodule clinic) returned to the initial face-to-face consultation model. 

The MDT meeting is attended by two consultants: a respiratory physician with a special interest in lung cancer and pulmonary nodules and a dedicated chest radiologist. The service is also linked with a database administrator. 

### 2.2. The Pathway

**Figure 1:**
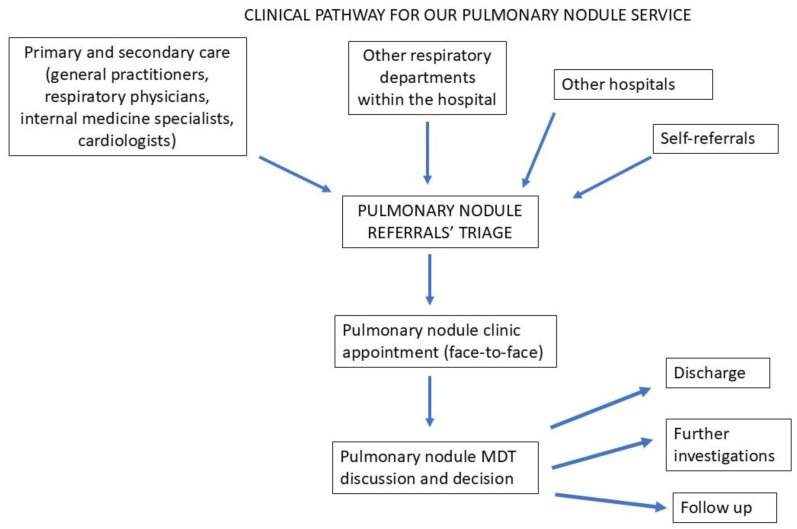
Our current clinical pathway for patients with pulmonary nodules.

Figure 1 shows the pathway that has been successfully implemented in our hospital.

**Figure 2:**
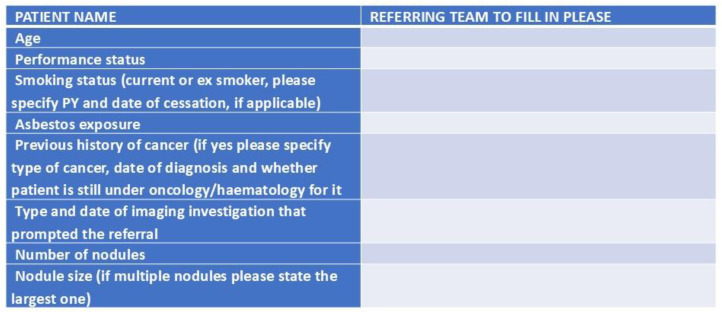
Proforma referral system for patients referred to the service.

The service receives referrals from a variety of sources: primary and secondary care (respiratory physicians, internal medicine specialists, cardiologists, general practitioners, etc.), other departments within the hospital and other hospitals and self-referrals. The latter is a unique feature in the Greek health system, where patients can self-refer to specialists based on the abnormal results of investigations they received from the diagnostic centres. In detail, the results of investigations requested by physicians are communicated directly to the patient, who can either return to the referring physician for a consultation or can self-refer to a specialist centre according to the result. Our service utilizes a referral proforma (Figure 2) that contains relevant clinical information and is filled in by the referring physician, or in the case of self-referral, by the receiving specialist. 

All referrals are triaged by the respiratory consultant with a special interest in lung cancer and pulmonary nodules. Patients are booked straight into the pulmonary nodule clinic where appropriate. Appropriate referrals are discussed in the pulmonary nodules MDT and inappropriate referrals are sent back to the referrer with documentation (i.e., why they are inappropriate for MDT discussion and nodule clinic follow-up) and proposed action plans (where appropriate).

As a safety net for patients who have undergone chest CT imaging in the emergency department of our hospital and incidental pulmonary nodules have been reported, the physicians in the emergency department contact the pulmonary nodule service and an overbooked pulmonary nodule clinic appointment is authorized to ensure that these patients are not lost in the system. 

All patients are reviewed in the clinic, and where appropriate, are discussed in the pulmonary nodules MDT, where they will be either discharged or referred for further testing or will be put under follow-up by the nodule service with serial low-dose chest CT scans for a certain period, depending on the type of nodule and patient’s past medical history. On some occasions, when a nodule has an increased risk stratification for malignancy, increases in size or changes its pattern on serial imaging, patients are offered a PET CT if that is deemed appropriate by the MDT. Should a patient require a PET CT, and its results raise concerns, then the patient pathway is automatically transferred to the lung cancer service (multidisciplinary thoracic oncology service—MTOS), where the patient will be seen in clinic with a view to discussing more invasive tests, e.g., tissue biopsy.

All outcomes are communicated to patients during the face-to-face clinic appointments. 

### 2.3. Referral Criteria

From April 2018 to April 2024, we used the BTS guidelines for the diagnostic work-up and follow-up of patients [19]. The choice of these guidelines was based on a multidisciplinary consensus between radiology, respiratory and thoracic surgery within our hospital, as well as the respective departments and nuclear medicine within the two biggest oncology hospitals in Athens and Piraeus. We accepted referrals of patients with pulmonary nodules that were new incidental findings and warranted consultation or were already under follow-up by other general respiratory services, or of patients who were new to the service, but were previously under the MTOS or other general respiratory clinics in primary/secondary/tertiary care, and had nodules >8 mm that were benign on PET CT criteria and/or on biopsy, in addition to patients with known nodules wishing to obtain a second opinion. Patients with a previous history of thoracic malignancy were excluded from the service and were followed up by MTOS as per standard processes, while patients with extrathoracic and/or hematological malignancy still under oncological follow-up were also excluded. Immunocompromised patients who were at risk of infection were also excluded; these patients were reviewed initially by the respective services, and their approach was based on the specific clinical situation. The service did not include patients with nodules that were detected during lung cancer screening, as our lung cancer screening clinic was established in 2022, and all nodules detected in that clinic are managed under a different streamlined pathway.

## 3. Results

We have retrospectively reviewed all previously collected data on patients referred to the pulmonary nodule service, discussed in the MDT and reviewed in the virtual clinic between April 2018 and April 2024. All patients data was fully anonymized.

During the reviewed period, 132 MDT meetings occurred, and 2203 patients were reviewed in the pulmonary nodules’ clinic (903 females, 1300 males, mean age 64 ± 19 years). Of those patients, 65% (1432/2203) were current smokers, 23% (506/2203) ex-smokers and the remaining 12% (264/2203) were lifelong nonsmokers. A history of asbestos exposure and TB was reported in 8% (176/2203) and 5% (110/2203), respectively, while 22% (485/2203) had a past medical history of extrathoracic/hematological malignancy, for which they were discharged from oncological follow-up. Emphysema was radiologically present in 72% (1586/2203) of all patients, and 75% of those (1189/1586) already had a confirmed diagnosis of chronic obstructive pulmonary disease (COPD) based on spirometry tests performed in primary or secondary care. Coronary calcification was identified in 32% (705/2203) of patients, and 78% of those (550/705) were already known to cardiology services. Table 1 shows the epidemiological data of patients discussed in the pulmonary nodules MDT meeting. 

**Figure 3:**
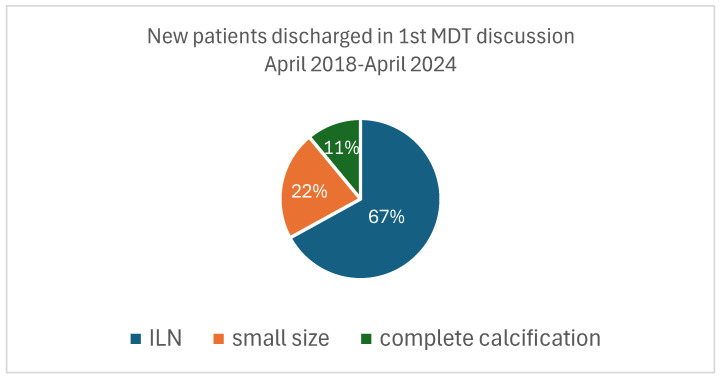
New patients discharged in the 1st MDT discussion. The ‘small size’ corresponds to nodules <5mm (BTS guidelines) [18].

Interestingly, 27% (368/1365) of new patients were discharged following their first clinic appointment, while the remaining patients continued to be under surveillance. It is of note that 67% (246/368) of these patients were discharged at their first clinic appointment because the ‘nodule’ that prompted the referral to our service was an intrapulmonary lymph node not warranting any further investigation or action. The remaining 22% (81 patients) were discharged as the nodule that prompted the referral was small (<5 mm), as per BTS criteria, and 11% (41 patients) were discharged due to having a single fully calcified lung nodule that required no further action (Figure 3). 

Among all patients, 11% (246/2203) were referred to the MTOS due to suspicious appearance or changes in their nodules that warranted further investigation, and from those, 37% were discharged (92/308) from MTOS. The lung cancer diagnosis rate was 7% (154/2203), and all patients were diagnosed in early-stage non-small-cell lung cancer, which was radically treated with surgery, therefore highlighting the importance of a dedicated pulmonary nodule service (PNS). Only 5.8% (9/154) of patients who underwent surgery had pN1 disease, which was confirmed by the surgical pathology report. Table 2 shows the nodule clinic and MDT meeting activity in terms of new and follow-up patients discussed, including the number of discharges as well as referrals to the MTOS. Table 3 shows the density of the surgically resected pulmonary nodules that were confirmed to be malignant in the final pathology. It is of note that the majority (68%) of surgically resected adenocarcinomas were part-solid nodules, while two of those initially presented as pure ground-glass nodules but developed an increasing solid component during radiological follow-up and were subsequently resected. As mentioned above, all patients underwent radical surgical resection; Table 4 presents the types of resections performed, and Table 5 presents the types of surgical modalities applied to patients per type of resection. It is of note that one patient with pT1aN0 underwent a lobectomy rather than a more lung-sparing parenchymal approach due to the central location of the pulmonary nodule adjacent to the pulmonary hilum. All patients presenting with pathologically confirmed N1 disease were subsequently offered systemic treatments based on their final pathology and molecular results. 

## 4. Discussion

### 4.1. The Problem of Incidental Pulmonary Nodules and the Need for Dedicated Structured Clinical Services

The management of incidental pulmonary nodules, reported by various imaging modalities outside a lung cancer screening programme, poses an additional challenge for clinical services which are already overwhelmed. It requires additional staff members, training and equipment, as well as dedicated clinical pathways [21,22]. Patients diagnosed with incidental pulmonary nodules outside a screening programme are usually investigated for symptoms irrelevant to the nodules, and they receive an unexpected diagnosis of an ‘incidentaloma’ which needs to be acted upon. This workload is projected to increase further with the wide implementation of national lung cancer screening programmes, which are anticipated to increase this challenge further. A non-negligible number of screening-detected pulmonary nodules will be found in addition to incidental nodules reported on CTPAs, chest CTs requested for various reasons, etc., as well as other incidental findings requiring further action. such as emphysema, coronary artery calcification, etc., [7,9,10,23]. 

We do not run a national lung cancer screening programme yet; therefore, our patients were diagnosed with an incidental pulmonary lung nodule while being investigated for a different clinical entity. Being aware of the need to offer structured clinical pathways, the standard operating procedure proposed by scientific organizations to the Greek health authorities involves a separate service for the management of lung cancer screening findings (e.g., nodules, emphysema, etc.), as screening candidates constitute different population from the ones investigated for an irrelevant symptom that are incidentally diagnosed with a pulmonary nodule [24]. 

In the US, from a population of over 321 million, it has been estimated that, each year, >1.5 million Americans will be found to have a lung nodule, and only 5% of them will be found to have lung cancer [25,26]. Moreover, all guidelines [16,17,18] recommend that the evaluation of pulmonary nodules needs to be performed in a timely manner to promptly identify the subgroups that are malignant. This means that a large proportion of patients will undergo repeat CT imaging, with implications for existing healthcare resources in terms of CT capacity and clinical services that will need to follow up these patients. To address this and ensure that the need for repeat CT imaging does not imply acute CT slots (i.e., slots for inpatients or accident and emergency), pulmonary nodule patients are usually scanned in dedicated afternoon slots within our hospital, or in private diagnostic centres where most of the scanning fee (90%) is covered by national health insurance [25]. This is the first established pulmonary nodule clinic and pathway in the national health system in Greece that offers a pragmatic multidisciplinary approach to address an increasing patient need and real-world data from this service. Our pathway offers a realistic approach to setting up a feasible service in a tertiary care setting with limited resources and a lack of additional funding. Pulmonary nodule services have been well established in the UK and the US [26,27]. On reflection, our pathway could include clinical nurse specialists in a more active role, managing these patients similarly to other institutions [28,29,30,31,32,33]; however, there is a national shortage of clinical nurse specialists who are currently assigned in acute medical/surgical services, and the Greek legislation does not allow registered nurses to lead nurse-led clinics as this is not included within their professional rights. Although specific recommendations have been published for incidental PNS in Greece [22], they are not aligned with the current processes and funding in the real clinical world. Significant restructuring and funding allocation are required to meet the proposed standards.

In countries with larger populations than Greece, the establishment of a dedicated clinical PNS would greatly benefit from the active involvement of clinical nurse specialists, who would take on patient management responsibilities—an approach already implemented in several of these countries, provided that this is in permitted by relevant legislation underlying nurses’ professional rights [28,29,30,31,32,33]. Additionally, the introduction of virtual clinics could help to alleviate the burden on overloaded services by optimizing time allocation based on clinical needs. This would improve efficiency for both patients and healthcare professionals, ensuring that in-person appointments are reserved for individuals with specific needs or those presenting with suspicious nodules requiring further investigation [27].

### 4.2. The Importance of Safety Netting and Appropriate Follow-Up

During the study period (April 2018–April 2024), we reviewed 2203 patients. Prior to setting up the dedicated pulmonary nodule service, these patients would have physically attended respiratory or MTOS outpatient clinics in new or follow-up patient slots. The new service has managed to save these outpatient appointments from MTOS and general respiratory clinics, streamline the patients and improve overall capacity in overwhelmed services. 

Moreover, we have provided a sustainable safety net to ensure that patients are appropriately followed up for incidental pulmonary nodules and are not lost to follow-up. This has been previously recognized as an emerging issue, as patients with incidental pulmonary nodules often do not receive appropriate evaluation or follow-up, and they ‘fall off the radar’ [34,35]. Blagev et al. [36] reported that pulmonary nodules were found in 9.9% of 1000 CT pulmonary angiographies ordered in the emergency department; however, their follow-up was poor, as less than a third of those patients were appropriately followed according to the Fleischner Society guidelines at that point. The situation has been reported to be similar in ambulatory care setting; Callen et al. [37] performed a systematic review of 19 studies with evidence of the number of tests not followed up for patients attending ambulatory settings and reported that 35.7% of radiology studies did not have appropriate follow-up from the ordering provider. It has been recognized that specific resources and structures are required to ensure that patients are appropriately followed up, and a formal process would significantly facilitate this [31,38,39,40,41]. In the US, the extent to which formal processes and structures have been applied nationally is unclear, and the American Thoracic Society has recommended more research in this area [12,42]. The situation in Europe is similarly unclear, and remains to be defined. With this background, our dedicated pulmonary nodule service has provided a formal process which ensures that all these nodules are appropriately followed up, and to our knowledge, this is the first study providing a pathway trialled in a pragmatic clinical setting; admin support and an updated nodule database are essential for this purpose, to ensure there is appropriate tracking system support [31]. 

### 4.3. Dedicated Specialized Personnel Improves Patient Management

In this service, we have discharged 27% of new referrals during the first pulmonary nodule clinic and MDT discussion due to a small nodule size not requiring follow-up, the presence of intrapulmonary lymph nodes (ILNs) that were reported as nodules or the absence of nodules. This highlights the importance of a dedicated chest radiologist reporting these scans and the importance of a specialized multidisciplinary team approach to the evaluation of these cases [43,44,45]. In a busy hospital setting, CT imaging is reported by radiology specialist registrars (i.e., senior trainees) and consultant radiologists, some of whom may have a special interest in chest radiology. Our hospital is a national referral centre for respiratory medicine, and therefore, our radiologists have a special interest in chest radiology. It is of note that, for the CTs of these new patients who were discharged after the first MDT meeting discussion, they were not initially reported by a dedicated chest radiologist. In addition to that, a non-negligible amount of these cases were intrapulmonary lymph nodes which were reported as nodules. Had it not been for a multidisciplinary team meeting approach in the presence of a dedicated radiologist and a respiratory physician with expertise in pulmonary nodules and lung cancer, these patients with ILNs would have entered a vicious circle of surveillance CT scans for a period of at least 2 years, and this would result in inappropriate use of resources, unnecessary patient anxiety and exposure to radiation. To our knowledge, this has not been thoroughly addressed in the literature on incidental nodules. Woloshin et al. [39] highlighted the importance of an enhanced radiology report for patients with incidental pulmonary nodules. The authors performed an internet-based survey of clinicians in one academic medical centre that preferred reports with management recommendations, and consequently, their response in terms of clinical management of these patients was improved. However, it is not clearly stated whether the radiologists reporting these scans were chest radiologists or not. Overall, inappropriate radiologist recommendations have been found to be the strongest predictor of care that follows guidelines inconsistently [35,36]. Radiologist recommendations have been found to be inconsistent with guidelines in up to 17.8% of cases [35], whereas in national surveys, it has been reported that 39–73% of radiologists had non-concordance with guidelines regarding follow-up recommendations [46,47]. Consequently, this poses uncertainty to the clinicians who will be asked to manage these patients with incidental pulmonary nodules based on the radiology report. This is where a specialized multidisciplinary team (MDT) meeting adds value and overcomes these obstacles [48]. In our service, recommendations for clinical management come through the dedicated pulmonary nodule MDT meeting, where all clinical and radiological aspects are discussed prior to a recommendation for further investigations, follow-up or discharge based on the guidelines. 

Our experience has shown that in a real clinical setting with overstretched resources, it would be very challenging to have all chest imaging reported by dedicated chest radiologists, especially in general hospitals. The pan-European shortage of chest radiologists is a reality [49]. Our hospital is a national referral centre for respiratory medicine; therefore, it is a hub for chest radiology expertise, and its clinical network collaboration with two oncology centres, where consolidated chest radiology expertise also exists, means that it holds an ideal position amidst the current challenges [50].

### 4.4. Pulmonary Nodules’ Density and Radical Treatment

In our cohort, all patients with pulmonary nodules initially assessed as suspicious by the specialized MDT underwent surgery with curative intent. The majority of patients with a pathologically confirmed adenocarcinoma (68%) presented with a subsolid nodule that was surgically resected. Among this majority, two patients initially presented with a pure ground-glass nodule that, on radiological follow-up, developed an increasing solid component, and was therefore surgically resected, confirming lung adenocarcinoma. The preference for a subsolid pattern in lung adenocarcinoma pathology is well-documented in the literature [51,52]. Given their distinct radiological evolution, these nodules necessitate careful clinical decision-making strategies. The solid density has been prevalent in squamous, adenosquanous large cell carcinomas and in typical carcinoids, which is consistent with the published data [14,16,17,18].

All patients were diagnosed in the early stages of lung cancer and were offered surgery with radical intent as the first course of action. The vast majority of patients with pT1a lung cancer underwent lung parenchyma-sparing surgery (wedge resection, segmentectomy), while patients with pT1b and pT1c mostly underwent lobectomies. Over 60% of surgeries per type of resection (wedge, segmentectomy, lobectomy) were performed with VATS. To the best of our knowledge, this is the first manuscript reporting on the type of resection and surgical modality for incidentally detected pulmonary nodules within a dedicated PNS and outside a lung cancer screening programme, which serves different populations. In our opinion, these results cannot be directly compared with malignant nodules detected within a screening programme as our patients underwent the initial chest CT imaging to investigate other underlying symptoms and comorbidities rather than being asymptomatic high-risk individuals participating in a screening programme [53].

### 4.5. Additional Radiological Findings Beyond Incidental Pulmonary Nodules 

Emphysema was radiologically present in 72% of our patients, and 75% of those already had a confirmed diagnosis of COPD from primary or secondary care, thus leaving 17.8% of all patients with radiological evidence of emphysema but no previous spirometry to assess subsequent obstruction. In these patients, an obstructive pattern was confirmed with a spirometry at a later stage. The only relevant available data regarding undiagnosed COPD come from lung cancer screening cohorts, where almost 19.7% of participants had undiagnosed COPD [54,55]. Patients with screen-detected nodules seem to present with similar percentages of undiagnosed COPD to our patients with incidentally identified nodules. In both studies, patients share similar smoking intensity habits. Coronary calcification was identified in 32% of our patients and 78% of those who were already known to cardiology services and had received treatment. This alludes to the fact that 7.7% of all patients remain undiagnosed in the community while having a coronary calcification in their LDCT. The only relevant available evidence comes from the lung cancer screening populations worldwide, rather than populations with incidentally detected pulmonary nodules [56]. Patients with screen-detected nodules mostly (55%) had undiagnosed coronary artery disease that was incidentally reported on the LDCT, which prompted a specialist referral. In our case, only 7.7% of all patients were undiagnosed, and this may be explained by the fact that our patients are a different cohort than the patients selected for lung cancer screening, who are clinically healthy [12]. They have been symptomatic, and therefore have been offered various investigations, including the imaging tests which led to the pulmonary nodule diagnosis. To the best of our knowledge, this is the first paper reporting data from this group of patients. The lung cancer detection rate in our patients is 7%, which is more than the lung cancer detection rate in screening (1–2%, ranging up to 4.5% in some cases) [57,58]. This may be explained by the fact that our population is generally older, and they have higher percentages of confirmed obstructive lung disease, which contributes to lung cancer formation. Patients diagnosed with lung cancer through the pulmonary nodule service were at early disease stages and underwent treatment with radical intent. All patients with suspicious pulmonary nodules were discussed in the lung cancer MDT to consolidate a tailored treatment plan according to their needs and medical history [22,27,48,59,60].

## 5. Conclusions

Our PNS offers a pragmatic approach that has been trialled in a busy clinical setting with limited resources. It is feasible in tertiary care, and it addresses an increasing patient need. Multidisciplinary involvement is of vital importance to ensure adherence to guidelines and appropriate case-by-case management, whereas robust administrative support is required to ensure that patients are appropriately tracked and not lost to follow-up. The increasing number of imaging investigations offered to patients and the implementation of LDCT screening are a reality; therefore, the development of dedicated pulmonary services is required to ensure the appropriate management of both groups of patients. The development of formal tracking systems and national/European registries will assist in the collection and audit of data and can have a significant impact on the development of future clinical guidelines and clinical research.

## Figures and Tables

**Table 1:** Characteristics of patients with pulmonary nodules under follow-up. * (TB: tuberculosis).

Characteristics	Number of Patients (%)
Sex	
Female	903 (41%)
Male	1300 (59%)
Mean age (years)	64 ± 19
Smoking habit	
Current smoker	1432 (65%)
Ex-smoker	507 (23%)
Lifelong non-smoker	264 (12%)
History of extrathoracic cancer (discharged from relevant services)	
Yes	485 (22%)
No	1718 (78%)
Asbestos exposure	
Yes	176 (8%)
No	2027 (92%)
Don’t know	-
History of TB *	
Yes	110 (5%)
No	2093 (95%)
Radiological evidence of emphysema	
Yes	1586 (72%)
No	617 (28%)
Radiological evidence of coronary calcification	
Yes	705 (32%)
No	1498 (68%)

**Table 2:** Pulmonary nodules clinic and MDT activity.

MDTs April 2018–April 2024	132
Patients	
Total number of patients discussed	2203
New patients	1365 (62%)
Follow-up patients	838 (38%)
Patients with solid nodules	1851 (84%)
Patients with subsolid nodules	352 (16%)
Discharges	
New patients discharged in the first clinic appointment	368/1365 (27%)
Intrapulmonary lymph nodes	246/368 (67%)
Small size	81/368 (22%)
Calcified nodule	41/368 (11%)
Referral	
Patients referred to MTOS	246/2203 (11%)
Patients referred to MTOS and discharged	92/246 (37%)
Lung cancer diagnosis rate	154/2203 (7%)
Adenocarcinoma	100/154 (65%)
Squamous cell carcinoma	46/154 (29.8%)
Typical carcinoid	1/154 (0.65%)
Large cell carcinoma	3/154 (1.95%)
Adenosquamous cell carcinoma	4/154 (2.6%)
Stage I at diagnosis (T1a-c N0, eighth TNM edition)	145/154 (94.15%)
Stage II at diagnosis (T1a-c N1, eighth TNM edition)	9/154 (5.85%)

**Table 3:** The final status of diagnosis for resected pulmonary nodules (*N* = 154) classified by their density.

Histological Diagnosis	Nodule Density		
	Solid	Part-Solid	Pure Ground-Glass
Adenocarcinoma (100/154)	32 (32%)	68 (68%)	0
Squamous cell carcinoma (46/154)	45 (98%)	1 (2%)	0
Typical carcinoid (1/154)	1 (100%)	0	0
Large cell carcinoma (3/154)	3 (100%)	0	0
Adenosquamous cell carcinoma (4/154)	3 (75%)	1 (25%)	0

**Table 4:** Types of surgical resection offered.

	N (100%)	Type of Resection		
Pathology Staging		Wedge Resection	Segmentectomy	Lobectomy
pT1aN0	38	20/38 (52.6%)	17/38 (44.8%)	1/38 (2.6%)
pT1bN0	78	0	29 (37.2%)	49 (62.8%)
pT1cN0	29	0	0	29 (100%)
pT1aN1	1	0	1 (100%)	0
pT1bN1	2	0	1 (50%)	1 (50%)
pT1cN1	6	0	0	6 (100%)

**Table 5:** Types of surgery and surgical modalities offered.

	Surgical Modality			
Type of Resection N		VATS (Video-Assisted Thoracic Surgery)	Open Thoracotomy	VATS Converted to Open Thoracotomy
Wedge	20	13 (65%)	5 (25%)	2 (10%)
Segmentectomy	48	30 (62.5%)	13 (27%)	5 (10.5%)
Lobectomy	86	54 (62.8%)	25 (29%)	7 (8.2%)

## Data Availability

The original contributions presented in this study are included in the article. Further inquiries can be directed to the corresponding author.

## Abbreviations

The following abbreviations are used in this manuscript:

LDCT	Low dose computed tomography
PNS	Pulmonary nodule service
MDT	Multidisciplinary team
COPD	Chronic obstructive pulmonary disease
MTOS	Multidisciplinary thoracic oncology service
TB	Tuberculosis
